# Incidence and mortality of breast cancer among adolescent and young adult women worldwide in 2022 and projections for 2040: Implications for surgery

**DOI:** 10.1016/j.breast.2026.104953

**Published:** 2026-10-29

**Authors:** Freddy Houéhanou Rodrigue Gnangnon, Bérénice Dossa, Issimouha Dille, Rodrigue Allodji, Benjamin O Anderson, Sophie Pilleron

**Affiliations:** a Université Paris-Saclay, Gustave Roussy, Inserm, UVSQ, Center for Research in Epidemiology and Population Health – Unit 1018, EpiRad (Radiation Epidemiology team), Villejuif, F-94805, France; b Division of Surgical Oncology, Faculty of Health Sciences, University of Abomey-Calavi, Cotonou, Republic of Benin; c Laboratory of Epidemiology of Chronic and Neurological Diseases, University of Abomey-Calavi, Cotonou, Benin; d World Health Organization Regional Office for Africa, Brazzaville, Republic of the Congo; e Departments of Surgery and Global Health Medicine, University of Washington, Seattle, WA, USA; f Ageing, Cancer, and Disparities Research Unit, Department of Precision Health, Luxembourg Institute of Health, 1A-B, rue Thomas Edison, Strassen, 1445, Luxembourg

**Keywords:** Breast cancer, adolescents and young adults, Incidence, Mortality, GLOBOCAN, Sub-Saharan Africa, Global disparities, Surgery

## Abstract

**Background:**

Breast cancer remains a major public health concern worldwide and is the most common malignancy among women. Although it predominantly affects women over 50 years, a growing number of cases occur in adolescents and young adults (AYA; aged 15–39 years). In low- and middle-Human Development Index (HDI) countries, particularly in sub-Saharan Africa, this group faces disproportionately high mortality rates.

**Methods:**

Number of new cases, deaths, and age-standardized rates (ASR) per 100,000 women aged 15–39 years in 2022 were extracted from the Global Cancer Observatory (GLOBOCAN) database. Data were analyzed for 185 countries and aggregated by the six United Nations regions. Projections to 2040 were estimated by applying 2022 age-specific rates to UN national population forecasts, assuming stable cancer risk and mortality patterns over time.

**Results:**

In 2022, an estimated 245,827 new cases of breast cancer occurred among AYA women, representing 10.7% of all breast cancer cases. Asia accounted for the largest number of incident cases (49% of the global total), while Africa showed the highest mortality rates (6.2 per 100,000 AYA). At the national level, the proportion of AYA cases ranged from 43.6% in The Gambia to 3.3% in Finland. By 2040, the number of breast cancer cases among AYA women is projected to increase by 7.6% globally, with Africa showing an approximately 57% increase in the numbers of incident cases and deaths.

**Conclusion:**

The rising burden of breast cancer among AYA women highlights a critical need to strengthen health systems in low- and middle-HDI countries. Our findings may help inform health system planning in regions where the burden of breast cancer among AYA women is projected to increase. They support consideration of context-appropriate strategies to improve early diagnosis, expand access to multidisciplinary cancer care, and strengthen surgical capacity, including oncoplastic and reconstructive surgery, where appropriate.

## Highlights

• AYA breast cancer represents >10% of cases and 7.3% of deaths worldwide.
• Major disparities: highest incidence in Asia, highest mortality in Africa.
• Sub-Saharan Africa faces disproportionate burden and poor survival.
• By 2040, the number of new breast cancer cases and deaths among AYA women in Africa may both rise by approximately 57%.
• High maternal mortality leads to significant intergenerational impact.

## Graphical abstract

**Figure:**
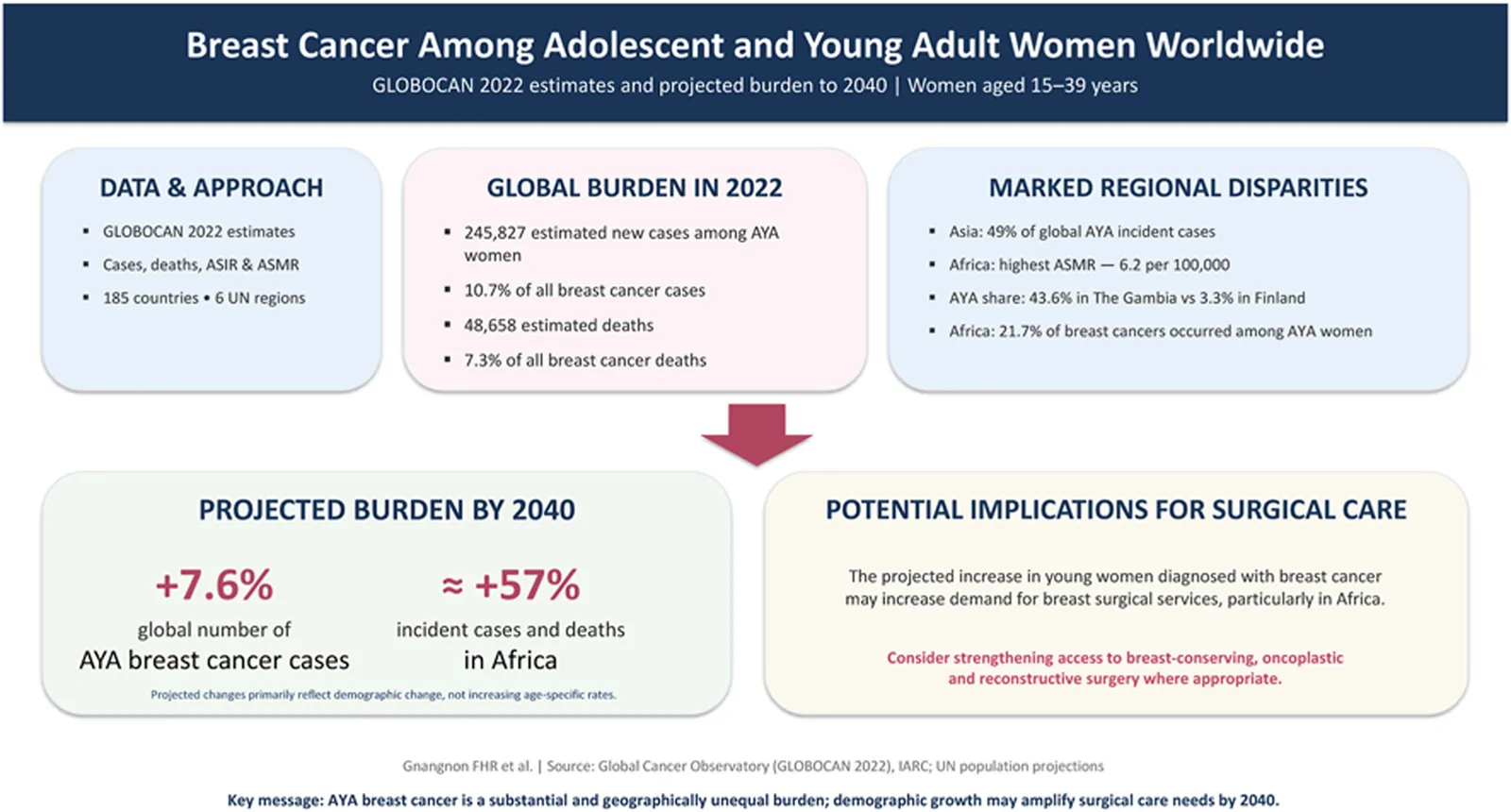

## 1 Introduction

Breast cancer remains a major public health concern worldwide. In 2022, an estimated 2.3 million new cases were diagnosed among women, and 670,000 deaths were attributed to the disease [1]. Across all contexts, regardless of economic development or health system capacity, it remains the most common cancer among women [2]. Over the past decades, a significant increase in incidence has been observed among young women [3].

Adolescents and young adults (AYA), defined as women aged 15 to 39 years according to the *National Cancer Institute* [4], are particularly affected. Countries with a low Human Development Index (HDI), especially in sub-Saharan Africa (SSA) show concerning rates of incidence and mortality, despite progress in detection and treatment.

Breast cancer in AYA women is characterized by more aggressive forms: advanced stages at diagnosis, unfavorable tumor phenotypes, a higher risk of recurrence, and limited therapeutic options [1], [2]. This aggressiveness often leads to complex surgical management, with many patients requiring mastectomy because of advanced disease at diagnosis.

Among AYA, these surgical interventions may have a major psychosocial impact, affecting body image, femininity, sexuality, and psychological well-being [4]. At an age marked by concerns related to fertility, motherhood, and professional integration, mastectomy can become a profoundly destabilizing life event [5], [6].

In resource-limited settings, diagnostic delays, the absence of multidisciplinary tumor boards, and restricted access to radiotherapy further aggravate the burden of surgical morbidity. The burden of breast cancer among AYA women appears particularly critical in sub-Saharan Africa, where limited diagnostic, surgical, and radiotherapy capacity contributes to disproportionately high mortality. These structural constraints, combined with younger population demographics, amplify the regional impact of the disease and justify focused analysis. Moreover, breast cancer-related deaths at a young age have a dramatic social impact: in sub-Saharan Africa, for every 100 deaths among women under 50 years, an estimated 203 orphans are generated, with country-specific variations from 189 in Nigeria to 247 in Zambia [7].

Although many studies have described the epidemiology of breast cancer in the general population, few have focused specifically on AYA women [8]; the available data remain fragmented and geographically limited, preventing a comprehensive understanding of the global burden of the disease in this age group [9], [10].

This study addresses this gap by providing a global and updated descriptive analysis of the incidence and mortality of breast cancer among AYA women in 2022, with projections for 2040, aiming to inform strategies for prevention, early diagnosis, and surgical management tailored to this specific population.

## 2 Methods

Data on breast cancer incidence and mortality for 2022 were extracted from the Global Cancer Observatory (GLOBOCAN) database, including the estimated number of new cases and deaths, as well as truncated age-standardized rates (ASR) per 100,000 women aged 15–39 years. These data, compiled and regularly updated by the International Agency for Research on Cancer (IARC), allow for reliable international comparisons. Age standardization was performed using the world standard population developed by Segi et al. and revised by Doll et al. [3].

GLOBOCAN provides modeled estimates rather than directly observed nationwide data for all countries. Where available and considered of sufficient quality, estimates are based on population-based cancer registry data from individual countries. In countries lacking reliable population-based cancer registry data, estimates are modeled using available national mortality statistics or extrapolated from neighboring countries or regions assumed to have comparable epidemiological profiles. The use of such modeling and extrapolation methods may introduce additional uncertainty into the estimates.

Data were compiled for 185 countries and grouped according to the six United Nations regions: Africa, Asia, Europe, Latin America and the Caribbean, North America, and Oceania.

To comprehensively describe the burden of breast cancer among AYA women, three complementary epidemiological measures were reported: (i) the estimated absolute number of incident cases and deaths, (ii) the estimated proportion of all breast cancer cases occurring among AYA women, and (iii) truncated age-standardized incidence and mortality rates (ASIR and ASMR). These indicators are derived from the GLOBOCAN 2022 database and therefore represent modeled estimates based on the best available cancer registry data, mortality statistics, and statistical modeling methods, rather than directly observed nationwide data for all countries. While absolute numbers reflect the overall burden of disease and proportions illustrate the relative contribution of AYA women among all breast cancer cases, ASIR and ASMR were used as the primary measures for comparisons across countries and regions because they account for differences in population age structure.

Projections for 2040 were derived by applying the age-specific estimated rates from 2022 to the United Nations national population projections. Consequently, the projections estimate the expected numbers of new breast cancer cases and deaths under the assumption that age-specific incidence and mortality rates remain constant over time [11]. Therefore, projected changes primarily reflect demographic changes, including population growth and shifts in age structure, rather than changes in the underlying risk of breast cancer.

Maps and Figures presented in the manuscript are extracted from the GCO website.

## 3 Results

### 3.1 Breast cancer incidence among adolescents and young adults (AYA)

In 2022, an estimated 245,827 new cases of breast cancer were diagnosed among AYA worldwide, representing 10.7% of all breast cancer cases across all age groups (Table 1).

**Table 1:** Number of cases and age-standardized incidence rates (ASIR) of breast cancer among women aged 15–39 years in 2022, by region and subregion.

Regions	Subregions	Number of cases	(ASIR per 100,000)
**Africa**		43,121	16.0
	Eastern Africa	11726	13.1
	Central Africa	4443	13.3
	Northern Africa	13696	25.2
	Southern Africa	1912	11.2
	Western Africa	11344	15.0

**Asia**		120,218	12.0
	Eastern Asia	42,003	12.5
	South-Central Asia	44,200	9.7
	South-Eastern Asia	23,064	15.4
	Western Asia	10,951	17.6

**Europe**		34,188	23.3
	Eastern Europe	11,910	18.7
	Northern Europe	4674	22.1
	Southern Europe	7541	29.1
	Western Europe	10,063	28.0

**Latin America and the Caribbean**		28,121	19.0
	Caribbean	1767	19.4
	Central America	6010	14.6
	South America	20,344	20.7

**Northern America**		18,092	24.1

**Oceania**		2087	23.0
	Australia/New Zealand	1601	24.5
	Melanesia	446	19.4
	Micronesia	9	8.2
	Polynesia	31	22.9
**World**		**245,827**	**14.9**

Among the six United Nations regions, the lowest proportions of all breast cancer cases occurring among AYA women were observed in North America (5.9%), Europe (6.1%), and Oceania (7.3%), whereas the highest proportions of all breast cancer cases occurring among AYA women were recorded in Africa (21.7%), Latin America and the Caribbean (12.8%), and Asia (12.2%). At the national level, The Gambia reported the highest proportion of breast cancer cases occurring among AYA women (43.6%), while Finland had the lowest (3.3%).

In absolute numbers, Asia accounted for nearly half of all global cases (120,218; 49%), followed by Africa (43,121; 18%), Europe (34,188; 14%), and Latin America and the Caribbean (28,121; 11%). North America (18,092; 7%) and Oceania (2087; 1%) had the smallest case numbers.

Regarding age-standardized incidence rates (ASIR), the highest values were observed in North America (24.1 per 100,000 AYA), followed by Europe (23.3) and Oceania (23.0). Africa showed an intermediate rate (16.0 per 100,000 AYA), whereas Asia recorded the lowest (12.0 per 100,000 AYA). These global variations are illustrated in Fig. 1, which maps the age-truncated standardized incidence rates (ASIR) of breast cancer among AYA women worldwide.

**Fig. 1:**
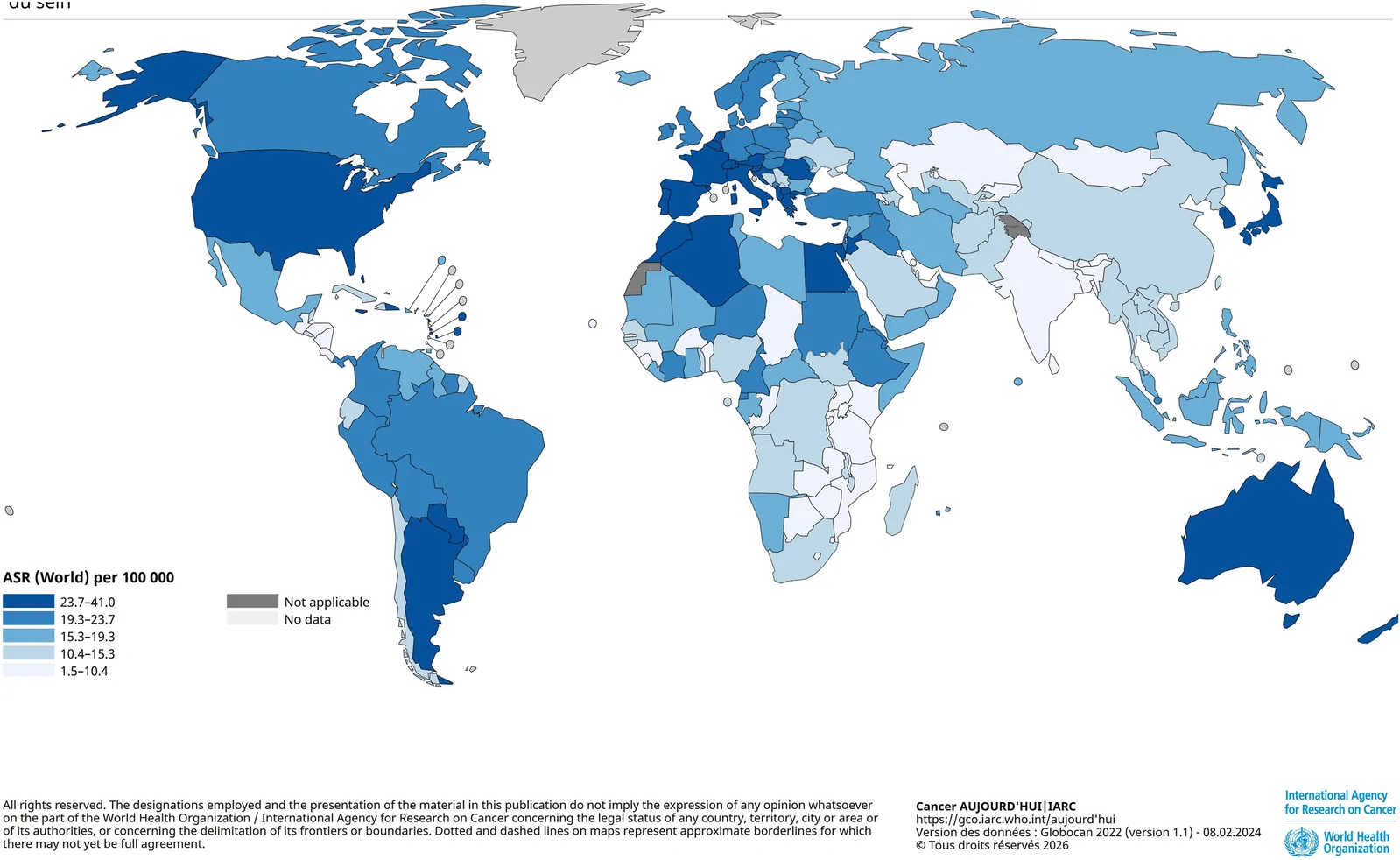
Age-truncated standardized incidence rates (ASIR) of breast cancer among adolescent and young adult women (AYAs) worldwide in 2022. Source: Global Cancer Observatory (GLOBOCAN 2022), International Agency for Research on Cancer (IARC).

### 3.2 Breast cancer mortality among AYA

In 2022, the global number of deaths from breast cancer among AYA women was estimated at 48,658, representing 7.3% of all breast cancer deaths across all age groups (Table 2).

**Table 2:** Number of deaths and age-standardized mortality rates (ASMR) of breast cancer among women aged 15–39 years in 2022, by region and subregion.

Regions	Subregions	Number of deaths	(ASMR per 100,000)
**Africa**		**16,574**	**6.2**
	Eastern Africa	5340	6.0
	Central Africa	2043	6.1
	Northern Africa	3561	6.5
	Southern Africa	546	3.2
	Western Africa	5084	6.8
**Asia**		**23,171**	**2.3**
	Eastern Asia	3932	1.1
	South-Central Asia	12,040	2.6
	South-Eastern Asia	4686	3.1
	Western Asia	2513	4.0
**Europe**		**3136**	**2.1**
	Eastern Europe	1419	2.1
	Northern Europe	409	1.9
	Southern Europe	503	1.8
	Western Europe	805	2.2
**Latin America and the Caribbean**		4091	2.7
	Caribbean	409	4.4
	Central America	830	2.0
	South America	2852	2.8
**Northern America**		1391	1.8
**Oceania**		295	3.3
	Australia/New Zealand	109	1.6
	Melanesia	180	7.8
	Micronesia	0	0.0
	Polynesia	6	4.3
**World**		**48,658**	**2.9**

The age-standardized mortality rate (ASMR) varied considerably by region, ranging from 1.8 per 100,000 AYA in North America to 6.2 per 100,000 AYA in Africa.

Asia accounted for the largest number of deaths (23,171; 47.6% of the global total), followed by Africa (16,574; 34.1%), Latin America and the Caribbean (4091; 8.4%), and Europe (3136; 6.4%). North America (1391; 2.9%) and Oceania (295; 0.6%) recorded the lowest number of deaths. At the national level, disparities were also marked: Chad had the highest proportion of all breast cancer deaths occurring among AYA women (37.9%), whereas Guam recorded none.

Regional differences in ASMR are further illustrated in Fig. 2, showing the age-truncated standardized mortality rates for breast cancer among AYA women worldwide.

**Fig. 2:**
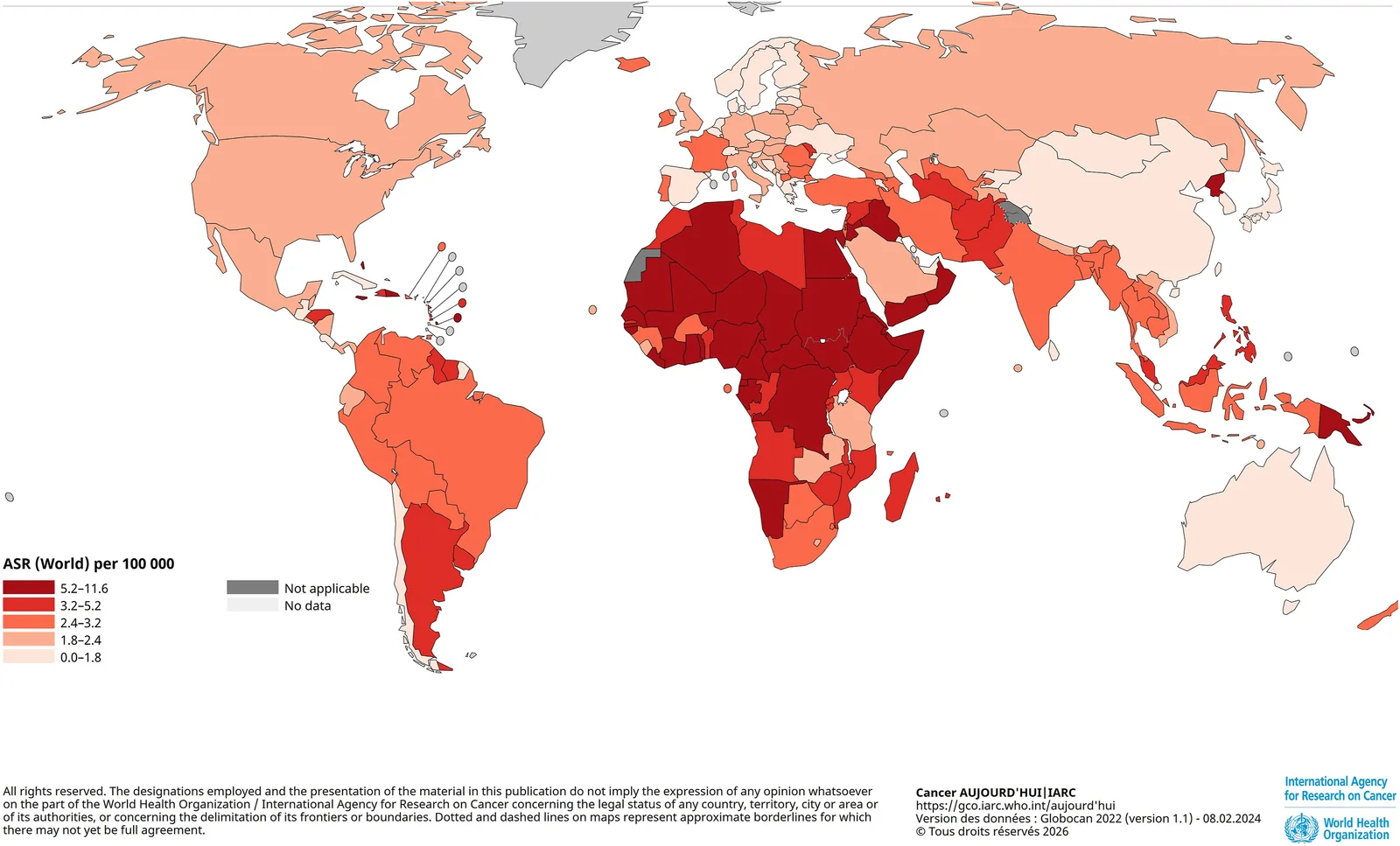
Age-truncated standardized mortality rates (ASMR) of breast cancer among adolescent and young adult women (AYAs) worldwide in 2022. Source: Global Cancer Observatory (GLOBOCAN 2022), International Agency for Research on Cancer (IARC).

### 3.3 Projected burden of breast cancer among AYA women by 2040

Based on demographic projections and assuming stable age-specific incidence rates, the number of new breast cancer cases among AYA women could increase to approximately 265,000 by 2040, representing an overall rise of about 7–8% compared to 2022 (Fig. 3). Regional patterns suggest that Asia is projected to remain the region with the largest number of incident cases, whereas Africa may experience the most pronounced increase (approximately +57%), largely driven by demographic expansion.

**Fig. 3:**
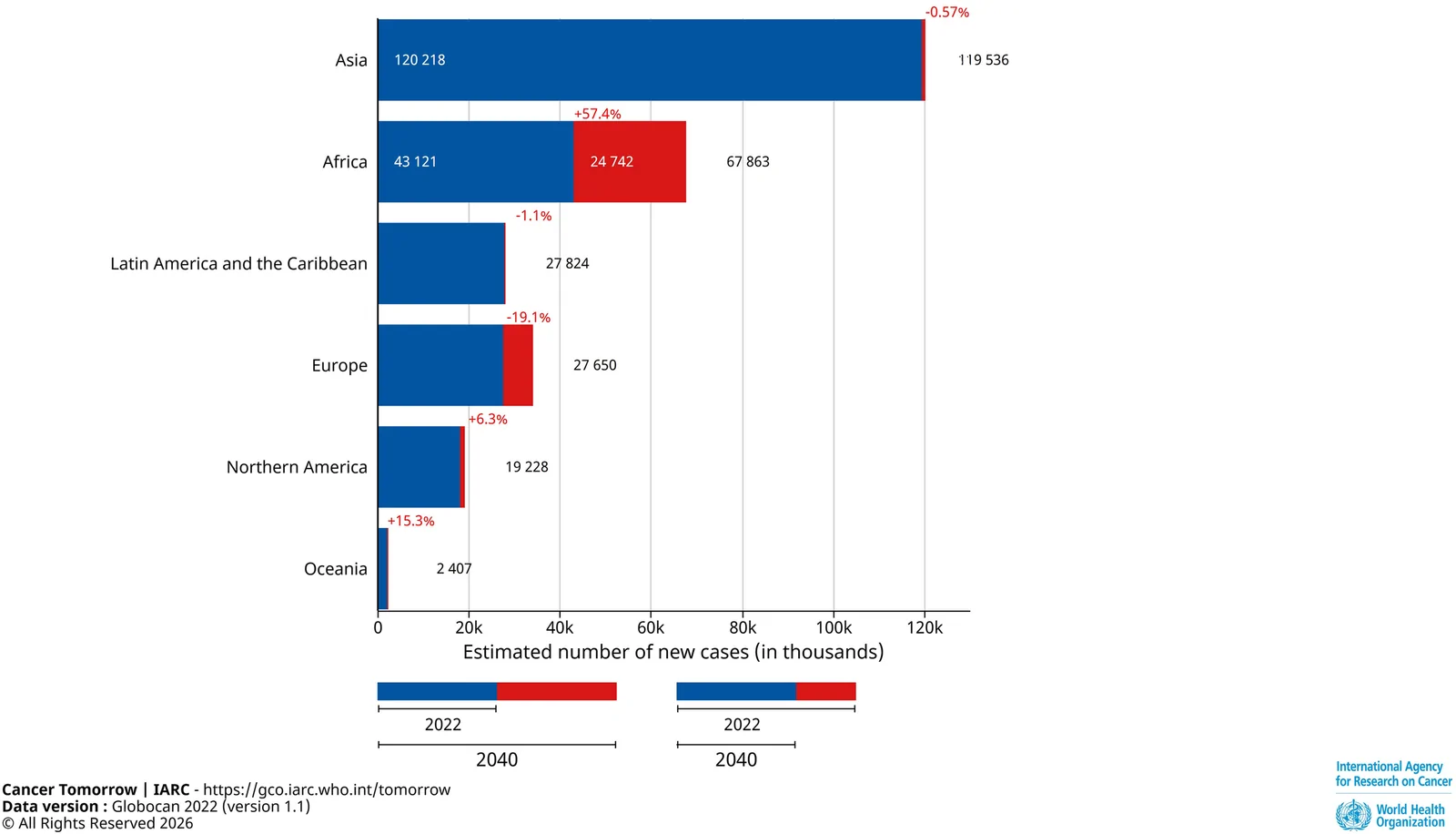
Projected number of new breast cancer cases among women aged 15–39 years (AYAs) worldwide by 2040. Source: Global Cancer Observatory – Cancer Tomorrow (GLOBOCAN 2022), International Agency for Research on Cancer (IARC).

In parallel, the total number of deaths among AYA women could rise to approximately 57,000 by 2040, corresponding to an increase of around 18% compared with 2022 (Fig. 4). Africa is projected to experience the largest relative increase in the number of deaths (approximately +57%), while Europe and Asia may experience relative declines in absolute deaths.

**Fig. 4:**
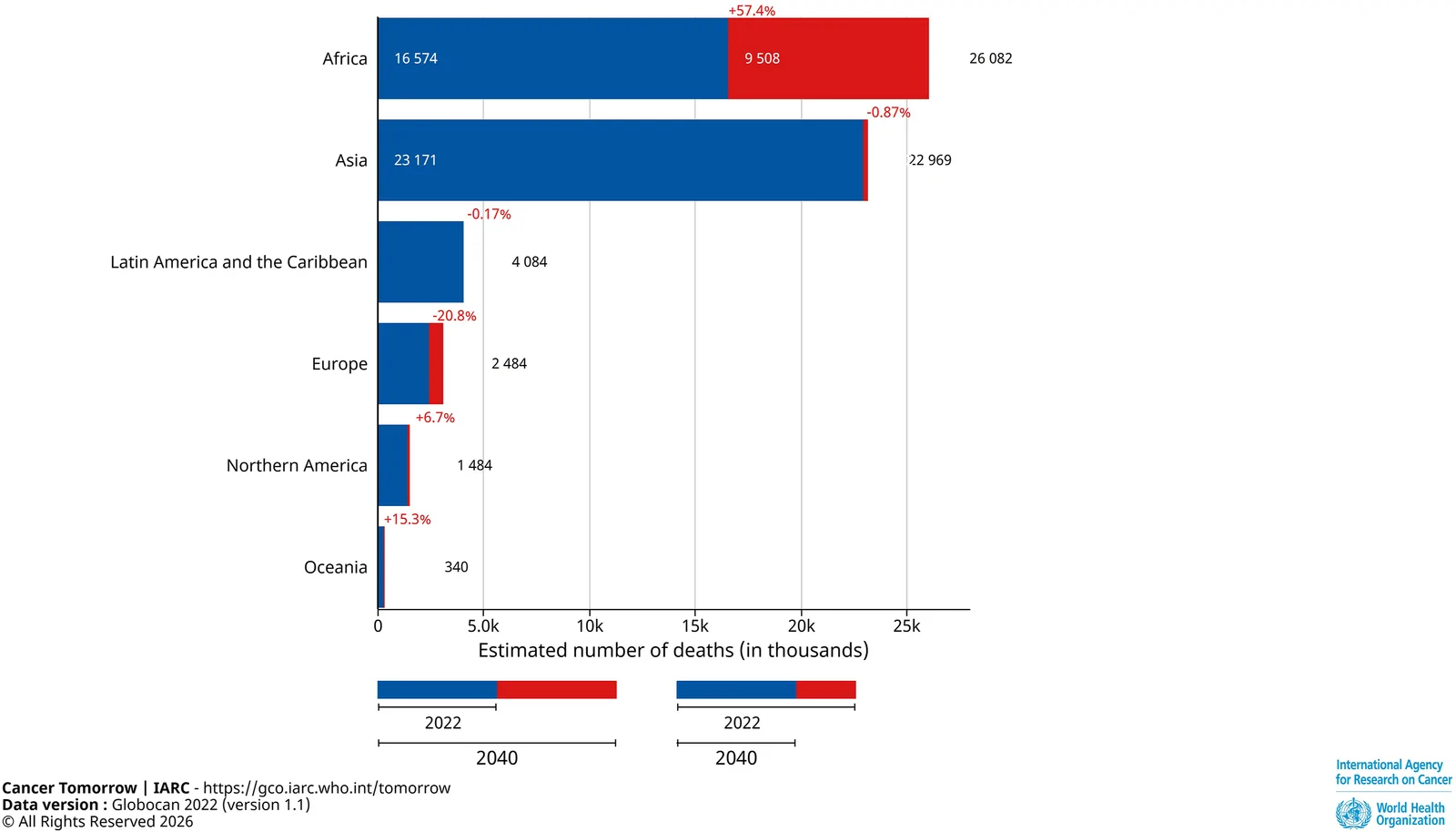
Projected number of breast cancer deaths among women aged 15–39 years worldwide by 2040. Source: Global Cancer Observatory – Cancer Tomorrow (GLOBOCAN 2022), International Agency for Research on Cancer (IARC).

These estimates should be interpreted as indicative demographic trends rather than precise predictive figures, given the modeled nature of GLOBOCAN data and the heterogeneity of cancer control systems worldwide.

## 4 Discussion

### 4.1 Main findings

This study highlights a substantial global burden of breast cancer among AYA women. In 2022, this group accounted for more than 10% of all new breast cancer cases, and 7.3% of global breast cancer mortality all ages combined. The largest number of incident cases was observed in Asia, while the highest age-standardized mortality rates occurred in Africa. By 2040, the global number of incident cases is projected to increase modestly (approximately 7–8%), Africa is projected to experience the largest increase (approximately 57%) in the number of breast cancer cases and deaths among AYA women. This increase is driven primarily by projected demographic changes under the assumption of stable age-specific incidence and mortality rates. Beyond the individual dimension, breast cancer mortality among AYA has major societal consequences.

In Africa, for every 100 deaths from breast cancer in women under 50, an estimated more than 200 orphans are generated, reflecting the humanitarian and economic impact of this disease [7]. The number of maternal orphans is closely linked to premature mortality among women under 50 years. Socio-economically disadvantaged groups, who also experience lower breast cancer survival, are more likely to generate a greater number of maternal orphans, thereby perpetuating a cycle of social and economic disadvantage [12]. This effect may be further amplified in sub-Saharan Africa, where fertility rates remain the highest globally. Notably, Africa is the only region where fertility still exceeds the global replacement level, with an average of approximately 4.7 children per woman [12]. These findings highlight the intergenerational consequences of cancer, particularly among young populations facing poor survival outcomes.

### 4.2 Definition and characteristics of breast cancer in AYA

The definition of AYA varies across contexts, directly influencing clinical management and care organization. In the Netherlands, for instance, patients aged 18–39 years are classified as AYA, as those under 18 are treated in pediatric oncology centers, while adults (≥18 years) are managed in general hospitals [13]. This distinction underlines the need for specific care pathways tailored to this age group, which often faces unique psychosocial and fertility-related challenges.

Biologically and clinically, breast cancer in AYA is characterized by more aggressive forms and later diagnosis. This population is generally excluded from organized screening programs, leading to delayed detection [14], [15]. Tumors are often diagnosed with higher histologic grade, and from unfavorable subtypes, such as triple-negative and HER2-positive breast cancer [16], [17]. A considerable proportion of cases are associated with germline mutations (BRCA1, TP53) and with distinct tumor micro-environmental features that may influence negatively treatment response [18]. These factors help explain the overall lower survival probabilities among AYA compared with older women [18], [19].

### 4.3 Geographical disparities in incidence and mortality

Our results reveal pronounced regional heterogeneity. The highest proportions of all breast cancer cases occurring among AYA women were observed in Africa (with The Gambia reporting 43.6% of cases in this age group) and Latin America and the Caribbean, in contrast to much lower proportions in Europe (Finland, 3.3%) and North America. These differences primarily reflect demographic variations: in countries with a low or medium Human Development Index (HDI), younger population structures (median age 18–20 years vs. 40–45 years in high-income countries) mechanically increase the share of AYA among all breast cancer cases. More broadly, three main hypotheses can explain this distribution [20].

1. A cohort effect, due to the larger proportion of young women in low- and middle-income populations.
2. Potential biological differences across populations.
3. Incomplete recording of cancers in older age groups in low-HDI countries, leading to overestimation of the relative proportion among AYA.

In 2022, Africa had the highest age-standardized breast cancer mortality rate among AYA women, at 6.2 deaths per 100,000 women per year, compared with 2.1 per 100,000 in Europe. In Chad, approximately 38% of all breast cancer deaths were estimated to occur among AYA women. The high burden of breast cancer mortality among young women in sub-Saharan Africa may be partly explained by delayed diagnosis and limited access to effective locoregional and systemic treatments [4]. Differences in tumor biology, including a potentially higher prevalence of triple-negative breast cancer in some sub-Saharan African populations [21], [22] may also contribute to these mortality disparities. This aggressive molecular subtype is associated with a poorer prognosis and, when diagnosed at locally advanced or metastatic stages, may require innovative systemic therapies, such as immunotherapy, in eligible patients. Given the higher frequency of late-stage diagnosis in sub-Saharan Africa [21], [23], limited access to these costly treatments may further contribute to unfavorable outcomes. However, the contribution of these biological and therapeutic factors to regional mortality disparities was not directly assessed in the present study. Conversely, young women in high-income countries benefit from broader access to expensive targeted and immunotherapies, breast-conserving surgery, and reconstructive procedures [24].

### 4.4 Potential surgical implications of the projected burden

Although our study was not designed to evaluate surgical interventions, the projected increase in the number of young women diagnosed with breast cancer, particularly in Africa, has potential implications for surgical care planning. The projected increase in the number of AYA women diagnosed with breast cancer is expected to increase the demand for breast surgical services. In this population, surgical management presents unique oncologic and psychosocial challenges. In low-resource settings, advanced stage at diagnosis frequently necessitates radical mastectomy because of delayed diagnosis and limited access to postoperative radiotherapy, which remains essential for breast-conserving treatment [25], [26].

The psychological and social consequences of such procedures are profound, including body image alteration, loss of femininity, sexual dysfunction, and social marginalization [27], [28]. The absence or high cost of breast reconstruction exacerbates this distress and hinders social reintegration [29].

Consequently, the projected increase in the number of AYA breast cancer cases may translate into a growing need for access to breast-conserving surgery, oncoplastic techniques, and reconstructive services, particularly in regions where the projected burden is expected to increase substantially.

### 4.5 Public health perspectives and planning

Projections to 2040 indicate a rise of the breast cancer burden among AYA, particularly in Africa. These trends may have implications for health system planning, including consideration of:

• Strengthening population-based cancer registries in low-HDI countries for accurate burden estimation;
• Adapting and disseminating screening guidelines to include younger high-risk women, as implemented in the United States;
• Developing specialized surgical capacities, especially in breast-conserving, oncoplastic, and reconstructive surgery;
• Expanding access to radiotherapy, chemotherapy, immunotherapy, and supportive care particularly for triple-negative breast cancer, which is more common among young women in sub-Saharan Africa.

### 4.6 Limitations and perspectives

The use of GLOBOCAN data, although allowing for standardized international comparisons, presents certain limitations. These are model-based estimates, compensating for the absence of cancer registries in several countries. In such contexts, underreporting of cases, limited mortality data, especially in low-HDI countries, and variable data quality may introduce bias and affect the accuracy of estimates. Therefore, estimates should be interpreted with caution, particularly in low-income countries with limited cancer registration systems.

It is also important to note that GLOBOCAN data are not individual factual records, but rather estimates derived from multiple sources (cancer registries, vital statistics, and national surveys), the reliability of which varies by region.

Moreover, projections to 2040 assume stable cancer risk and mortality patterns over time. This assumption may misrepresent the true future burden if major changes occur in screening policies, treatment access, or demographic transitions.

Despite these limitations, this study provides a global and updated overview of the burden of breast cancer among adolescents and young women worldwide.

## 5 Conclusion

Our findings highlight the increasing burden of breast cancer among adolescents and young women (AYA) and the considerable disparities across World regions. The projected number of breast cancer cases and deaths among AYA women is expected to increase substantially, particularly in Africa, largely because of demographic changes rather than increases in age-specific incidence or mortality rates. These findings may help inform health system planning in low- and middle-HDI countries, particularly in Africa, where the projected increase in the number of cases and deaths is greatest.

Particular attention should be given to the adaptation of clinical guidelines, the improvement of access to radiotherapy and innovative systemic therapies, and the development of oncoplastic and reconstructive surgery for young breast cancer patients.

## CRediT authorship contribution statement

**Freddy Houéhanou Rodrigue Gnangnon:** Writing – original draft, Methodology, Conceptualization. **Bérénice Dossa:** Writing – original draft, Investigation, Formal analysis, Data curation. **Issimouha Dille:** Writing – original draft, Validation, Supervision, Methodology. **Rodrigue Allodji:** Writing – original draft, Validation. **Benjamin O Anderson:** Writing – original draft, Validation, Supervision. **Sophie Pilleron:** Writing – original draft, Validation, Supervision, Methodology, Formal analysis, Conceptualization.

## Ethics approval and consent to participate

Not applicable.

## Consent for publication

Not applicable.

## Availability of data and materials

The datasets used and/or analyzed during the current study are publicly available on the Global Cancer Observatory (GLOBOCAN) website.

## Financial declaration

This study received no funding.

## Note on the use of artificial intelligence

During the preparation of this article, the generative artificial intelligence tool ChatGPT (GPT-5, OpenAI; accessed November 2025) was used to assist with translation and linguistic reformulation of specific paragraphs. Its use was limited to language editing, and all content was reviewed, validated, and finalized by the authors.

## Declaration of competing interest

The authors declare that they have no known competing financial interests or personal relationships that could have appeared to influence the work reported in this paper.
